# Asparagus breeding: Future research needs for sustainable production

**DOI:** 10.3389/fpls.2023.1148312

**Published:** 2023-03-27

**Authors:** Daniel Drost

**Affiliations:** Department of Plants, Soils and Climate, Utah State University, Logan, UT, United States

**Keywords:** temperature, mechanical harvest, productivity, yield, cultivars (varieties)

## Abstract

Productivity in asparagus (*Asparagus officinalis* L.) is determined in part by (1) the selection of superior, adaptive genetics, (2) matching the selected genetics to the production environment, and (3) managing the crop production system in ways to maximize harvest potential that are sustainable, profitable, and efficient. Over the last 100 years, a considerable effort by asparagus researchers has gone into breeding superior genetic lines, testing those in numerous locations, and studying how asparagus responds to a multitude of inputs (fertilizers, irrigation, fungicides, herbicides, insecticides). Farmers worldwide have benefited from all of these improvements. However, as we look to the future, we need to change our research approaches to deal with widely accepted limitations to asparagus growth that if left unanswered will further erode the long-term sustainability and profitability of the crop. In addition, there is a growing need for increased mechanization to offset labor needs. To effectively harvest asparagus, new plant types with more predictable spear emergence patterns need to be bred. This paper will briefly review the historic content of asparagus research and open a discussion on how to refocus international research efforts to breed superior plant materials to meet the challenges of the future.

## 1 Introduction

Asparagus (*Asparagus officinalis* L.) is an economically important perennial horticultural crop. It is grown worldwide in a variety of climatic conditions. Major asparagus production regions (key countries; area of production) include East Asia (China, Japan; 100,915 ha), Europe (Germany, Spain, France, Italy; 58,590 ha), North America (Mexico, the USA, Canada; 43,835 ha), South America (Peru, Chile; 36,585 ha), and Australasia (Australia, New Zealand; 2,036 ha) (FAOSTAT; www.fao.org/faostat; 2022). Each region and nation has its own unique challenges. Environments vary greatly from subtropical and tropical regions with year-round harvest potential to temperate areas limited to spring harvest systems. Production approaches are unique and include such things as mother-stalk culture, spear-forcing systems, green or white spear production, and multiple harvests each year, to name a few. Given the limited genetics available, cultivars need to be widely adapted to a variety of climates, soils, and management practices.

Productivity varies greatly around the world and is tied to the climatic conditions of the production area, production approaches, and harvest techniques. Peru and Mexico have the highest spear production at 11,670 and 9,430 kg ha^−1^, respectively (FAOSTAT; www.fao.org/faostat; 2022). In contrast, average yields in Asia (5,344 kg ha^−1^), Europe (5,010 kg ha^−1^), North America (4,146 kg ha^−1^), and Australasia (4,247 kg ha^−1^) are about half of the yield of high production areas. The high yields in Peru and Mexico are due to excellent climatic conditions during harvest and fern growth periods, multiple harvest cycles per year, and production near the equator, which favors year-round production potential. In the other production regions, productivity is limited by the temperate climate, which forces a spring (March–June) spear harvest, a summer (July–October) fern period, and a winter (November–March) dormant season, with the length of the harvest window depending of the region’s climatic conditions. Yields in temperature regions are generally lower than in more tropical regions as frost events may damage early spears, cool soil and air temperatures often slow spear initiation and growth rates, and the length of the spear harvest season needs to be balanced with fern development and root carbohydrate recharge after harvest. In temperate production regions of Europe, plastic mulches are widely used to regulate soil temperatures to improve spear productivity. High tunnels are also being integrated into green production systems to generate early spear emergence or for protection against disease. Other management practices may be integrated into the production system in the future to modify the environment and therefore alter the production period and/or enhance yield.

Asparagus is a long-lived perennial vegetable grown for its tender shoots (spears). The underground part is the crown, and the above-ground foliage is called the fern (Robb, 1984; Drost, 2020). The crown consists of a lateral rhizome or stem, numerous adventitious fleshy roots at the apices of the rhizome, and many buds collectively known as bud clusters (Tiedjens, 1924). The above-ground fern has many individual stems, which grow from the buds on the rhizome. Stems are called spears during harvest or ferns when fully elongated and developed (Robb, 1984; Drost, 2020). Fern generally grows up to 1–2 m tall. The storage roots grow for several years (Weaver and Bruner, 1927), extend out from the rhizome several meters (Drost and Wilson, 2003; Drost, 2023), and store long-chain sugars (Shelton and Lacy, 1980; Haynes, 1987; Wilson et al., 2008) that are used to grow the next crop of spears and ferns. Growing off the storage roots are small, short-feeding roots that absorb water and nutrients (Weaver and Bruner, 1927).

For those who grow asparagus, the growth of spears and the vigor of fern are used to assess present and future crop performance (Robb, 1984; Falloon and Nikoloff, 1986; Wolyn, 1993; Knaflewski, 1994; Knaflewski et al., 2012). Since asparagus is harvested for a decade or more, assessing productivity in research trials is costly and time-consuming. Being able to identify the correlation between early yield (yields before a crop comes into full production) and cumulative yields (total production over the crop’s life span) is important to reduce research costs. Total yields and fern stalk counts were highly correlated between consecutive years (Coyne, 1967; Moon, 1976; Knaflewski et al., 2012), and marketable yields from the first two harvest seasons were related to cumulative yields after 4 to 6 years (Bussell et al., 1987). However, second- to fourth-season yields were not associated with those from years 9 and 10 (Falloon and Nikoloff, 1986). Total harvested yield may (Moon, 1976) or may not be (Wolyn, 1993) associated with total fern number during the summer but a stalk (fern) index considering stalk count and diameter (Ellison and Scheer, 1959; Ellison et al., 1960b) or stalk size (Wolyn, 1993) may be highly correlated with yield. Yield evaluation by judging aboveground growth (spears or fern) often overestimates productivity. When evaluating asparagus plant performance, it varies widely among test locations, cultivars, level of plant care, and age, and generally, the cause of the variation cannot be identified. Wilson et al. (2008) argued that performance variability can be reduced and yield estimates increased by assessing supplementary plant information like carbohydrate content in addition to fern-based assessments.

Like all crops, asparagus growth is complex and primarily driven by energy capture (photosynthesis) (Bai and Kelly, 1999; Faville et al., 1999), with the assimilated carbon stored in the fleshy storage roots. Asparagus genetics is also important since cultivars are often selected for unique environments (Lin, 1979; Falloon and Nikoloff, 1982; Garrison et al., 1999; Motoki et al., 2008). There is some evidence of cultivar differences in photosynthesis and yield (Benson and Takatori, 1980; Woolley et al., 1996; Bai and Kelly, 1999; Faville et al., 1999; Guo et al., 2002). Therefore, connecting genotypes to environments can lead to productivity improvements. In most management systems, asparagus productivity depends on fern, root, and bud growth that occurred the year before, usually several months earlier. In addition, seasonal stress events are compounded from year to year, as does the connection between performance and productivity. This makes it difficult to track how environmental and management factors affect the plant. For example, the link between when to stop harvesting or how much fertilizer, irrigation, or fungicide to apply during fern growth (Hartmann, 1985; Falloon and Grogan, 1991; Knaflewski, 1994; Brainard et al., 2019) and the resulting spear production several months, or years, later is confusing, and the correlation between these is weak. It is not the purpose of this paper to discuss how management practices impact performance, suffice it to say, events during the annual growth cycle, including spear, fern, and root growth, carbohydrate production, storage, and re-utilization are key to successful productivity (Wilson et al., 2008). Therefore, a deeper understanding of genetic adaptability, integration with environmental conditions, and how these can be used to improve crop management will be briefly discussed.

## 2 Asparagus breeding

Cultivated *Asparagus officinalis* has been reported to have a narrow genetic base (Knaflewski, 1996). Many but not all commercial asparagus varieties come from the Netherlands and are offspring of “Violet Dutch” (Geoffriau et al., 1992). Recent efforts in studying the genetic diversity and provenance of asparagus (Li et al., 2016; Chen et al., 2020; Amato et al., 2023) report that it was not possible to clearly group the studied accessions, and the origins of cultivated asparagus are clearly more complex than originally expressed (Knaflewski, 1996). This may be a limiting factor for further asparagus breeding improvements, or there may be yet undiscovered traits useful to modern asparagus production. An alternative approach is to introduce traits of interest from wild relatives of asparagus (Castro et al., 2013). Commercially, asparagus is seed propagated. Seeds from selected crosses are the common method of propagation (Ellison, 1986), and sometimes the parental selection is done vegetatively, through the division of a rhizome, to obtain clonal plants from the selected genotypes (Encina and Regalado, 2022). Crown divisions are expensive, may spread diseases (e.g., *Fusarium* sp.) to new plantings (Encina and Regalado, 2022), and may not be suitable for the selection of the traits of interest (Falloon and Nikoloff, 1986). Due to climate change, there is a growing demand by the asparagus sector for the release of new varieties having higher yields and better resistance/tolerance against biotic (e.g., pests and diseases) and abiotic (e.g., drought, arid/hot climate conditions) stresses.

*A. officinalis* is a dioecious species (Ellison, 1986), with a 1:1 male:female sex ratio in open-pollination conditions. Male plants have been shown to outperform female plants (Currence & Richardson, 1937; Yeager and Scott, 1938; Franken, 1970). Reported differences in performance between male and female plants were associated with improved longevity, better tolerance to diseases, limits on seed production, and improved carbohydrate manufacturing (Currence and Richardson, 1937; Yeager and Scott, 1938; Bouwkamp and McCully, 1972; Ellison, 1986; Lopez-Anido and Cointry, 2008). These benefits promote faster growth and higher yields. For all these reasons, there has been a concerted effort to identify and develop “all-male” cultivars.

Since there are no morphological differences between male and female plants (Kanno, 2014), asparagus breeders obtained male plants by self-crossing andromonoecious flowers of male plants (Sneep, 1953a; Sneep, 1953b). When andromonoecious plants are self-pollinated, the resulting offspring segregate as one female to three males. One of the males is homozygous for maleness and was classified as a “supermale.” These “supermales” are important for breeders and growers because crossing a “supermale” with any female yields only male plants (“all-male”) known to be superior to females for yield, longevity, and disease tolerance, and they did not produce seeds or berries (Hartmann, 1985; Ellison, 1986). “Lucullus,” the first commercial “all-male” variety, was developed from the crossing of andromonoecious plants (Sneep, 1953a). Ellison (1986) noted that “supermales” are rare in asparagus populations, often have poor agronomic traits, and require a long time to identify. Similar techniques were used to create the New Jersey series of “all-male” hybrids (Ellison and Kinelski, 1985; Ellison and Kinelski, 1986; Ellison et al., 1990). Alternative approaches are needed to obtain supermales from selected males with outstanding agronomic traits. The development of di-haploid males through *in vitro* culture techniques provided a solution (Doré, 1990), where the “all-male” cultivars were F1 hybrids and thus more uniform. The first F1 all-male hybrid asparagus was “Andreas” (Corriols et al., 1990).

Knaflewski (1996) charted the significant achievements in asparagus breeding, including the identification and selection of “Mary Washington” with its quality spears and rust disease resistance. Many present-day cultivars find their origins in Mary Washington, including many supermales. The asparagus breeding community continues to make genetic improvements by focusing on local adaptions to unique environments (Falloon, 1982; Falavigna et al., 1999) and through efforts to adapt cultivars to diverse environments (Ellison et al., 1990; González and del Pozo, 2002). In addition, they make selections for earliness (Ellison and Schermerhorn, 1958; Ellison et al., 1960a), emphasize high productivity (Ellison et al., 1990), focus on spear quality (Huyskes, 1959; Siomo, 2018), and identify disease resistance (Lewis and Shoemaker, 1964; BusselI and Ryan, 1974).

Continued use of new technologies to develop new asparagus genotypes will improve growers’ options for overcoming the challenges of climate change, new and persistent pests and diseases, the need for high-quality products, and sustainable productivity. To overcome the narrow genetic base in *A. officinalis*, the introduction of new genes from wild species of asparagus may help solve present crop limitations (Falavinga et al., 2008; Castro et al., 2013; Encina and Regalado, 2022). Given the potential outcomes from these potential new asparagus types, asparagus breeders need to work more closely with asparagus physiologists to ensure *Asparagus officinalis* cultivars integrated with landraces and/or wild species are truly better and able to tolerate the changing biotic and abiotic stresses commonly experienced.

Interspecific hybridization of cultivated asparagus with wild relatives may provide new genes possessing important agronomic traits and genetic resistance to diseases and abiotic stresses (González-Castanon and Falavigna, 2008). Some interspecific crosses among asparagus species have been successful (Thevenin, 1974; Ochiai et al., 2002; Ito et al., 2007), while other crosses were reported as unsuccessful (Marcellàn and Camadro, 1996). Ideally, the key findings ultimately will be whether these new interspecific hybrids contribute something to asparagus production systems. Historically, important productivity characteristics commonly screened for included high total yield, high-quality spears (tight tips; uniform shape), good spear thickness, and improved earliness (Van den Broeck and Boonen, 1990). In a comprehensive review of the preharvest factors affecting asparagus quality (Siomo, 2018), genetic factors, environmental conditions, and crop management approaches are all important when evaluating spear quality. Additional effort has gone into the identification of disease resistance, as many production areas have difficulty with a range of biotic conditions that limit crop productivity (Rameau and Bota, 1990; Falloon and Grogan, 1991). Furthermore, numerous studies have evaluated existing asparagus genetic resources for adaptions to varied production conditions (Garrison et al., 1999; Benson, 2002; Drost, 2002; González, 2006; Motoki et al., 2008; Paschold et al., 2008b) and have helped identify cultivars with broad and narrow adaptions. As the international asparagus industry copes with uncertainty, breeders of asparagus need to work toward finding solutions to climate adaptations and changing labor needs while continuing to develop new cultivars that integrate into these systems.

## 3 Environmental issues

For a perennial plant like asparagus, one of the key goals is that crop yield (spear production) be optimized for a particular environment. Rymon (1988) states that obtaining stable yields (consistency over time) first requires both “interdisciplinary viewpoints and cooperative efforts by different professionals” and that “optimal and maximum yield” are often distinct and separate goals. Naturally, there are multiple limitations within any production environment, and climate is one of the major controlling factors in determining plant productivity (Krug, 1996; Ernst and Krug, 1998). Given the changes occurring in climate worldwide (science.nasa.gov/earth-science/oceanography/ocean-earth-system/climate-variability), significant effort will be needed by asparagus researchers and practitioners to create innovative approaches to cope with climate variability without compromising yield goals. The rise in global temperature may not be the main problem, but the redistribution of heat over the Earth’s surface may create more or less favorable production areas. Some production regions will warm, while others will cool, and these changes, plus the shifts in rainfall patterns, could positively or negatively influence agricultural regions across the planet. While asparagus has specific environmental requirements, it is widely adapted to many different geographic environments (Desert, Mediterranean, Tropical, Continental, and Subarctic). Asparagus is considered to be heat, drought, salinity, and cold tolerant. Although these are very diverse environmental conditions, asparagus is classified as a cool season crop and the conditions for optimal productivity are 24°C–29°C day and 13°C–19°C night temperature, which favor high productivity and longevity (Swiader and Ware, 2002; Drost, 2020). At temperatures lower than the optimum, all growth-related events are slower (Culpepper and Moon, 1939a; Culpepper and Moon, 1939b; Bai and Kelly, 1999), while at temperatures higher than the optimum, growth and assimilation rates decrease significantly (Yen et al., 1992).

It is the combined effects of the environment (abiotic) interacting with soil and biotic factors that have the greatest impact on the development, growth, and ultimately the achievement of optimal yield for asparagus. In a series of papers over several years, (Krug, 1996; Krug, 1997; Krug, 1998; Krug, 1999a; Krug, 1999b) and Ernst and Krug (1998) explored how various climatic conditions affected the seasonal growth and development of asparagus. While this body of work provided a glimpse into some of the mechanisms for adapting to variations in temperature, further evaluation and exploration of these topics is required to understand asparagus adaptation to and integration into changing environments. Yield “stability” is a critical component of dealing with climate uncertainty and selecting cultivars for local environments (Olesen et al., 2011). Therefore, identifying the thing that creates year-to-year variability is essential if commercial asparagus operations are to be successful, stable, and sustainable. The primary goal of production and the most important factor of profitability is the yield, it is important to know the relationship between the magnitude of yield fluctuations and the environmental factors that influence productivity (Krug, 1997).

Temperature is one of the leading controllers for initiating asparagus growth. The minimum temperatures required to initiate spear growth vary widely, and the reported values range from a low of 4.4°C (Bouwkamp and McCully, 1972), 5.0°C (Kim and Sakiyam, 1989), 5.6°C (Lampert et al., 1980), and 7.2°C (Blumenfield et al., 1961), up to a high of 11.1°C (Culpepper and Moon, 1939a). Asparagus plants are grown in the field display distinct growth cycles in desert, Mediterranean, continental, and subarctic regions, including a period of dormancy over the colder winter months. There is considerable genotypic variation among asparagus cultivars in their response to prior chilling when exposed to different subsequent growing temperatures (Ku et al., 2007). Chilling decreased the number of days to bud break in “Apollo,” “Rutgers Beacon,” and “Jersey Giant,” but had no effect in “UC 157.” Given the wide range of genetic materials available and the deviations in temperatures reported to initiate growth, more research is needed to understand the variability in responses to dormancy induction and growth resumption for existing cultivars adapted to colder production regions.

In asparagus production regions with cold winter conditions, cold acclimation and winter hardiness are necessary for crop longevity. Late-season vegetative growth adversely influences future yield in asparagus (Bai and Kelly, 1999) as well as winter hardiness. Cold acclimation occurs in two stages (Krug, 1999a). First, short days and cool temperatures (10°C–20°C) cause carbohydrate accumulation and secondary compounds to form (Landry and Wolyn, 2011). When temperatures are below 0°C, freezing tolerance increases. For the cultivars “Guelph Millennium” and Jersey Giant, freezing tolerance differences may be due to differences in rhizome characteristics or fern senescence (Landry and Wolyn, 2011). Due to the lack of other studies, additional varietal screening of other cold-tolerant germplasm is needed to ensure adaptability for asparagus grown in cold production regions.

In tropical, Mediterranean, and desert asparagus production regions (warm to hot conditions seasonally or year-round), high temperatures can limit spear growth initiation, cause excessive spear elongation rates, or negatively influence the fern’s ability to produce carbohydrates if temperatures are excessive (Dufault, 1990). No spears emerged when soil temperatures were maintained above 35°C (Dean, 1999), but due to limited studies at extreme temperatures, this needs further evaluation. Once spears emerge, the rate of spear extension increases as temperature increases within the 10°C–30°C range (Culpepper and Moon, 1939a; Culpepper and Moon, 1939b; Nichols and Woolley, 1985; Kim and Sakiyam, 1989; Dean, 1999). However, at temperatures above 35°C, spear growth slowed significantly (Dufault, 1996; Dean, 1999). Slow growth rates at low or cool temperatures delay the harvest of individual spears and delay the initiation of the next spear in the bud cluster. This reduces the total yield within a given harvest period. As temperatures increase, spears grow faster and more are harvested, which increases yield potential (Dean, 1999; Wilson et al., 1999). However, at very high temperatures, spear quality issues (Poll, 1996; Heißner et al., 2006) arise (off colors, open tips, split spears, or spear curvature). Total spear number is not generally impacted by high temperatures, but yield decreases as the number of nonmarketable spears increases. Very little is known about varietal differences in cold or heat tolerance, and additional work is needed.

Asparagus fern architecture is conducive to high photosynthetic capacity (Downton and Torokfalvy, 1975; Bai and Kelly, 1999). There is minimal shading of leaves, light penetrates deeply into the canopy, and photosynthetic rates are high throughout the day. Photosynthetic rates for different genotypes are often different in many crops, including asparagus (Bai and Kelly, 1999; Faville et al., 1999; Guo et al., 2002), and this variability was positively correlated with yield. The photosynthetic response to temperature indicates that maximum rates occur near 20°C, and as temperature increases to 30°C, photosynthesis decreases (Inagaki et al., 1989). Guo et al. (2002) reported high fern sucrose concentrations in the afternoon suggesting that assimilation exceeds export, carbon accumulates in the cladophyll tissue, and/or may result in sugar buildup due to environmental conditions experienced by the plants (temperature or water stress). As noted, asparagus is a highly temperature-dependent plant that grows faster during the day than at night (Culpepper and Moon, 1939b; Robb, 1984; Yen et al., 1996). Future breeding efforts need to consider these during the selection process, and additional work is urgently required to address the shortfall in the understanding of temperature-dependent processes in asparagus.

Research shows there is a strong positive correlation between plant population, root biomass, root carbohydrate (CHO) levels, and spear yield (Wilson et al., 2008). Therefore, creating optimal growing conditions early in the life cycle of the asparagus bed will ensure that stands are maintained, root size is large, and yield is optimized for the conditions. The level of stored CHO in the root system is one indicator of yield potential. Wilson et al. (2002a) compared the crown to an “engine,” with its buds and storage roots, and the CHO stored in the roots as the “fuel” needed to drive spear and fern production. Root CHOs are the main elements needed for high productivity (Shelton and Lacy, 1980; Haynes, 1987; Pressman et al., 1993). Root CHO is known to fluctuate in a distinct pattern associated with the growth of spears during harvest and ferns later in the season (Drost, 2020). During harvest, CHO levels decrease slowly, and as fern develop and grow, root CHO content falls rapidly (Pressman et al., 1993; Wilson et al., 2002b; Wilson et al., 2008; Paschold et al., 2008a). Once the fern reaches maturity, root CHO recharge increases rapidly. If plants are overharvested and/or if other stresses occur during the year, CHO recovery is compromised and future yield is negatively impacted. Thus, knowledge of the level of CHO in the roots at distinct times during the seasonal growth of asparagus becomes a useful measure for assessing the condition of the crop. Researchers and growers around the world have been using various measures of root CHO to quantify asparagus productivity (Wilson et al., 2008; Paschold et al., 2008a; Romero-Vergel, 2023).

## 4 Revisiting asparagus root systems

Asparagus root average size and distribution differ among cultivars (Benson and Takatori, 1980; Pressman et al., 1993; Drost, 2023). We also know that root size depends on plant population (Wilson et al., 2008), on soil conditions (Reijmerink, 1973; Drost and Wilson, 2003), is influenced by cultural practices (Drost and Wilcox-Lee, 2000), and increases rapidly during the establishment years (Drost, 2008; Paschold et al., 2008a; Drost, 2023). Much of the effort by asparagus breeders has been to select for high productivity. Plants with large root systems have the capacity for high productivity because they have many bud clusters (thus many buds) and also have large root systems (high CHO storage capacity). It is surprising, however, that more effort has not gone into quantifying asparagus root systems. Goals for asparagus breeding programs, selection techniques for specific characteristics, and how to design breeding programs have been outlined by Ellison (1986). Given the research effort, only minor mentions were made of assessing “healthy root” systems in breeding for resistance. Given the time required to develop new hybrids, while screening for existing traits, some effort to assess root development would significantly improve our understanding of the plant.

Wilson et al. (2008) argued that measuring root biomass was “impractical” and “unnecessary,” even though they and others (Drost and Wilson, 2003; Paschold et al., 2008a) showed that root mass is correlated with productivity. Asparagus root structural biomass increases rapidly in young asparagus plants (Drost, 2023) and then changes quite slowly after the crop reaches maturity (Drost and Wilcox-Lee, 2000; Drost, 2012). Therefore, changes in root biomass may not cause yield variability unless something drastically alters the root system. Year-to-year yield differences are commonly due to abiotic or biotic effects rather than root size differences (Lin, 1979; Dufault, 1990; Drost and Wilson, 2003; Brainard, 2012; Brainard et al., 2019). The historic effort to breed for high yields (large root size) attempts to focus on creating storage capacity for CHO and nutrients and for the crown to initiate and develop lots of bud clusters and buds. However, recent work clearly illustrated that asparagus cultivars vary in root distribution patterns, which may be important when selecting germplasm for unique production conditions (Drost, 2023). Unless we know more about how asparagus root systems vary relative to above-ground fern, we are only getting half of the story.

## 5 Future needs for asparagus—challenges and changes

Asparagus producers worldwide are facing similar issues. While the global demand for asparagus is increasing (www.mordorintelligence.com/industry-reports/asparagus-market; accessed 7 January 2023), labor cost and availability are hindering farm operations in many production regions (Holst et al., 2008; Calvin and Martin, 2011). Therefore, there needs to be a renewed interest in mechanization to replace labor shortages (Arndt et al., 1997; Holst et al., 2008; Cembali and Hood, 2009). There are two kinds of asparagus harvesting machinery. Some machines harvest nonselectively, where all spears are cut during the harvest operation regardless of their quality, length, or other unique requirements. In contrast, selective machines harvest spears based on specific criteria (Zhang et al., 2020). Nonselective harvesters for green asparagus cut all spears regardless of length at or near the soil surface. Cut spears are grabbed and deposited in collection boxes, or due to the speed of forward motion, cut spears are flicked into the collection device. Nonselective harvesters for white asparagus cut all spears regardless of length just above the crown in the soil mound then transport both spears and soil onto a conveyor, before separating the spears from the soil by vibration. The soil is then re-mounded over the plant row.

Selective harvesters for green asparagus require advanced sensor data processing to identify individual spears of the appropriate length from fields of spears of many different lengths (Arndt et al., 1997; Leu et al., 2017; Kennedy et al., 2019). Spear identification is difficult in on-farm conditions where speed and accuracy are paramount. To be integrated into existing production systems, selective harvesters need to operate at speeds and efficiencies comparable to human labor and perform the task without damaging the harvested spear or injuring surrounding spears in the field that was not selected for harvest. Thus, harvesters require real-time (in the order of tens of milliseconds) perception and cognition of the spears, as well as high-speed actuators and robust mechanical designs (Kennedy et al., 2019). Given enough computational power and accurate GPS data, nonselected spears could be mapped for future harvest, which may speed up harvest operations. Predicting when spears achieve the appropriate length for harvest requires knowledge of spear growth rates in addition to position information.

Selective harvesters for white asparagus are more complex (Dong et al., 2011; Geyer, 2018). To automate the harvesting process for white asparagus, the row guidance system needs to maintain the integrity of the asparagus soil mound, and the spear harvesting device needs to identify the spear’s location, cut off the spear under the ground, and successfully extract it from the soil with minimal damage to the spear. The guidance part is easier to create, while much more engineering is needed to get the spear-cutting and extraction mechanisms to work efficiently (Geyer, 2018). Seyfried and Schoebel (2016) evaluated ground-penetrating radar as an alternative approach to sensing crown depth to obtain the optimal cutting height for nonselective harvesters. The approach would ensure that long spears would be cut while crown damage would be minimized. Regardless of the production systems (green or white asparagus), harvesting robots need to be fast, inexpensive, produce little losses, and be gentle with the spears being handled (Clary et al., 2007).

An added difficulty with mechanizing asparagus harvest is that the present cultivars have been selected for hand harvest and have plant characteristics that do not lend themselves to machine harvest. In hand harvest systems, desired characteristics include high total yield, excellent spear quality, good spear thickness, and improved earliness (Van den Broeck and Boonen, 1990). The selection criteria that are lacking include uniform and consistent spear emergence, a better knowledge of what triggers bud break and spear elongation, a good understanding of bud and bud cluster dominance, more uniform spear positioning in the planted row, spear growth regulation, and harvest termination details. While temperature is the major driver of spear initiation and spear elongation, daily and seasonal variations in temperature alter the growth rate of spears. As labor costs continue to rise, the need to mechanize harvesting will increase. Present asparagus production systems are inefficient, particularly when harvest aids are employed. Harvest speeds are determined by the slowest worker, or when productivity is high, worker speed slows down relative to others. Geyer (2018) noted this in white asparagus productions and reported waiting times ranged from 1.2 to 1.6 h ha^−1^ depending on if yields were high or low in the field.

While a high total yield is important, spear production within the bud clusters of asparagus plants is sequential rather than concentrated and is variable rather than uniform. Growing spears inhibit the growth of subsequent spears in individual clusters, and over time, spear diameter decreases along the cluster (Woolley et al., 2008). Factors that hasten bud size development during the bud initiation period of the previous (prior year) growth period (Haynes, 1987; Drost and Wilcox-Lee, 1997; Ku et al., 2007) and continued sizing of younger buds during the harvest period (Ku et al., 2007; Woolley et al., 2008) may promote spear size since thicker spears are produced from large buds. Bud development in the crown continues during the period of fern growth (Drost and Wilcox-Lee, 1997; Drost, 2020), and a portion of these buds will produce spears during the next harvest cycle. Bud break patterns are also controlled by apical dominance (Tiedjens, 1926; Robb, 1984; Drost, 2020), soil and air temperature (**Environmental issues**), root carbohydrate content (Shelton and Lacy, 1980; Wilson et al., 2008), and plant growth regulators (Mahotiere, 1976; Matsubara, 1980). Thus, asparagus spears are harvested daily, and when environmental conditions favor rapid growth, twice daily. Since spear growth is a temperature-dependent response, better knowledge of or prediction of spear growth would help in scheduling harvests.

The history of the mechanical harvesting of tomatoes provides an analogy for how to approach the mechanization of asparagus harvest. Initially, there were no tomato harvesters (all hand harvested), and few had any idea of what they would look like or how they might operate (Stevens and Ricks, 1986). However, those developing the system figured the plants would need to produce fruits with greater firmness (protection from machine damage) and with a very short fruit-set period (concentrated fruit ripening). Beginning with the small determinate cultivar “Red Top,” and through selective breeding and screening, the VF 145 lines were developed. These first mechanically harvested processing tomato cultivars allowed mechanization to dominate present-day processed tomato production.

In apple orchards, the primary objective is to grow trees that produce high-quality fruit with high productivity. Over time, tree forms have changed through a selection of different plant types (dwarfing rootstocks) and the adoption of various training and pruning approaches. Planting density has a stronger effect on fruit quality, growth, and light interception than training systems (shape) at the same spacing (Hampson et al., 2002; Robinson, 2007). Light distribution within the canopy is more important than total light interception with regard to fruit quality. Therefore, efforts to increase fruiting spurs, as opposed to vegetative shoots, have significantly improved plant performance (Lauri et al., 2004). More linear growth habits (spur or central leader type) optimize light capture while reducing the need for extensive training or pruning (Lauri et al., 2009). Therefore, tree selection (or manipulation) creates new possibilities in orchard management, is more sustainable, improves labor efficiency, and reduces inputs (fertilizer, water, pesticides, etc.) while ultimately enhancing productivity.

So how are tomatoes and apples related to asparagus? First, like tomato and apple, asparagus breeders need to identify new plant types (different crown architecture) that may be more conducive to mechanical harvesting. Historically, asparagus breeding has focused on the identification of all-male lines, disease resistances, high spear yields (large crowns; lots of bud clusters), and spear quality as ways to address productivity. Ideally, asparagus plants with a more centralized growth habit (fewer secondary bud clusters or single/limited axis of rhizome growth) would help concentrate maturity or overcome the uncertainty of where spears may emerge in the field. Current asparagus cultivars have highly differentiated rhizomes (high branching; big crown), similar to indeterminate tomatoes. Existing asparagus varieties have many bud clusters; clusters and buds are highly variable in size and number, thus there is the potential for high yields. As plants develop, growth progresses in too many directions and may be spread out, and bud break follows no real pattern or predictability. These plant types are less adapted to mechanical harvesting due to the randomness of spear position, timing, or growth. Presently, there is no known way to regulate the timing of spear emergence, and it is difficult to determine when or where spears will emerge. So, existing asparagus plant types and present-day asparagus harvesters are less compatible, and this reduces harvest efficiency and increases harvest costs. In theory, asparagus crowns with stronger apical dominance may have fewer secondary bud clusters or may suppress those bud clusters from growing spears. Plants with a centralized rhizome (if identified) could be organized, much like spur or spindle apples, into narrow rows (high plant populations) and oriented in rows with distinct arrangements (bud clusters positioned directionally), which would better fit mechanical harvest. This does not overcome the problem of regulating spear emergence but does regulate the field position of the spears. While fewer bud clusters (fewer buds) would reduce spear yield per plant, lower per-plant yields could be overcome by increasing plant populations. Asparagus breeders need to not only consider traditional selection metrics but also keep a lookout for the unusual or off-types that may fit into nontraditional production systems.

Agriculture (and asparagus) is facing a second crisis on top of the shortage of agricultural workers. This is the failure to replant fields due to high establishment costs and/or a shortage of suitable land. In the USA, asparagus acreage has decreased by 77% from 31,323 ha in 2000 to 7,284 ha in 2020 (FAOSTAT; www.fao.org/faostat; 2022). While in Australasia, asparagus acreage has decreased by 60% from 5,074 ha in 2000 to 2,036 ha in 2020. In Europe, acreage is up by 17% during the same period, but in South and Central America, acreage is up by 42% and 138%, respectively. High labor costs, limited labor availability, increasing competition, and low-profit margins are the primary contributors to the reduced interest in asparagus. Recently, Taguchi et al., 2018 and Taguchi and Motoki, 2022. described the “whole harvest cultivation method for 1-year-old plants” as a way to reduce costs, increase yields, and improve land use. Asparagus is planted in year 1, and all spears are harvested in year 2; once production fall, the field is tilled out, and the process begins again (Kabuno et al., 2018; Taguchi et al., 2018). Spear yields were reported to be higher than the mean yield for traditional systems. The use of this method significantly shortens the production cycle but requires the selection and/or breeding of fast-growing, high-yielding, and large spear-generating cultivars (Taguchi and Motoki, 2022). Some existing cultivars have been evaluated for “annual asparagus,” but more studies on high early yields for this system are required. An annual production system requires cultivars that are fast-growing, produce a few large bud clusters and buds, and manufacture and store large amounts of carbohydrates in a short 1-year period. In addition, the “whole harvest” system may function more effectively if plant growth in the first year can be enhanced by using plastic mulches (Frenz and Munz, 1968; Makus and Gonzalez, 1991; Jakše and Marši´c, 2005), low or high tunnels (Dyduch et al., 2014; Chen et al., 2022), or by changes in plant populations (Takatori et al., 1975; Bussell et al., 1997). Ideally, asparagus breeding improvements are needed for the optimization of production in this short production-cycled system. Key selection parameters are big fern, big buds, and large root systems that can be produced during the first year, as well as cold tolerance, low dormancy, and uniform sprouting (Taguchi et al., 2018).

## 6 Conclusions

Changing asparagus production systems for mechanical harvesting would require an additional understanding of apical dominance within the bud clusters, renewed efforts to synchronize spear emergence, and the need for asparagus breeders to look for and identify alternative plant types specifically for the machine harvest industry. Asparagus breeding has historically focused on high productivity. Bigger plants do produce higher yields, but they do this randomly. Creating simpler systems (fewer buds and bud clusters) and identifying alternative plant forms could simplify production without sacrificing productivity. While many regions of asparagus production with high acreage still rely on hand harvesting methods, architectural changes to the asparagus plant would and could benefit all asparagus production regions making harvest more efficient and cost-effective. Furthermore, while much is known about asparagus growth, further work on the regulation of spear growth can help with understanding the dynamics and timing of spear elongation. Through the combined efforts of breeders and physiologists, the changes described can improve asparagus productivity, adapt the plant to mechanization, and still maintain the level of productivity and quality needed to make the system sustainable and profitable.

## Author contributions

The author confirms being the sole contributor of this work and has approved it for publication.
